# The Substitution of Fishmeal with Yeast Culture in the Yellow Catfish (*Pelteobagrus fulvidraco*) Diet: Growth, Serum Biochemical Indices, and Intestinal and Hepatopancreatic Histology

**DOI:** 10.3390/ani14060869

**Published:** 2024-03-12

**Authors:** Hongfei Huang, Xiaoqin Li, Beibei Guo, Yugui Zhang, Xu Yang, Yan Liu, Xiangjun Leng

**Affiliations:** 1 National Demonstration Center for Experimental Fisheries Science Education, Shanghai Ocean University, Shanghai 201306, China; hfhuang1998@163.com (H.H.); xqli@shou.edu.cn (X.L.); kailincao2023@163.com (B.G.); z09vad@163.com (Y.Z.); 2 Centre for Research on Environmental Ecology and Fish Nutrition (CREEFN) of the Ministry of Agriculture, Shanghai Ocean University, Shanghai 201306, China; 3 Shanghai Collaborative Innovation for Aquatic Animal Genetics and Breeding, Shanghai Ocean University, Shanghai 201306, China; 4 Shanghai Yuanyao Biological Co., Ltd., Shanghai 200120, China; yangxu@jsyuanyao.com (X.Y.); liuyan@jsyuanyao.com (Y.L.)

**Keywords:** yellow catfish, yeast culture, growth performance, nutrient utilization, serum immune, serum antioxidant, intestinal histology, hepatopancreas histology

## Abstract

**Simple Summary:**

Soybean meal is the most widely used plant protein source, but its application in aquaculture is limited by the existence of anti-nutrient factors. Fermentation can degrade the anti-nutrient factors in soybean meal. Yeast culture is a complex fermentation product, which is composed of yeast cells, metabolites and fermentation medium formed by multi-stage fermentation. Yeast culture contains plenty of β-glucan and mannan oligosaccharides, which can promote the growth, intestinal health and immunity of aquatic animals. This study investigated the potential of yeast culture substituting for fishmeal in the diet of yellow catfish (*Pelteobagrus fulvidraco*). The results showed that in the practical diet containing 160 g/kg fishmeal, yeast culture could effectively replace 40 g/kg fishmeal without adverse effects on the growth performance, nutrient utilization, serum biochemical indices, and intestinal and hepatopancreas histology of yellow catfish.

**Abstract:**

Yeast culture is a complex fermentation product consisting of fermentation substrate, yeast cells and their metabolites. This study investigated the potential of yeast culture in replacing fishmeal in the diet of yellow catfish (*Pelteobagrus fulvidraco*). First, a basal diet was formulated to contain 160 g/kg fishmeal (CON), and then the dietary fishmeal was decreased to 120, 80, 40 and 0 g/kg via yeast culture inclusion, respectively, to form another four isonitrogenous and isolipidic diets (YC-12, YC-8, YC-4 and YC-0). Yellow catfish (3.00 ± 0.10 g) were fed with the above five diets with triplicates per treatment and 40 fish per replicate. After 8 weeks of feeding, the weight gain (WG), protein efficiency rate and protein retention in the YC-12 group and the feed conversion ratio (FCR) in the YC-12 and YC-8 groups showed no significant differences to the CON group (*p* > 0.05), but the WG in the YC-8, YC-4 and YC-0 groups was significantly lower, and the FCR in the YC-4 and YC-0 groups was significantly higher than in the CON group (*p* < 0.05). In terms of the whole-body composition, only the crude lipid content in the YC-0 group decreased significantly (*p* < 0.05). Compared with the CON group, the aspartate aminotransferase and alanine aminotransferase activities and D-lactic acid content in the YC-0 group were significantly increased, and the total cholesterol content was significantly reduced (*p* < 0.05). The activities of catalase, superoxide dismutase, and alkaline phosphatase, as well as the content of complement C3 and immunoglobulin M, were significantly increased, while the MDA content was significantly reduced in the YC-12 and YC-8 groups (*p* < 0.05). There were no significant differences in the intestinal amylase and lipase activity among all the groups (*p* > 0.05), while the trypsin activity in the YC-12 and YC-8 groups, as well as the diamine oxidase in the YC-4 and YC-0 groups, were significantly higher than those in the CON group (*p* < 0.05). In the intestine histology, there was a significant decrease in the intestinal villus height in the YC-4 and YC-0 groups as well as in the villus width in the YC-0 group (*p* < 0.05). In the hepatopancreas histology, lipid droplets appeared in the YC-4 and YC-0 groups, and severe cell vacuolation was observed in the YC-0 group. As a summary, in a practical diet containing 160 g/kg fishmeal, yeast culture can effectively replace 40 g/kg fishmeal without negatively affecting the growth performance, nutrient utilization, serum immune and antioxidant, intestinal and hepatopancreas histology of yellow catfish.

## 1. Introduction

Fishmeal is an excellent protein source in aquafeed. However, due to the rapid development of aquaculture, the production of fishmeal could not meet the demand for aquafeed, resulting in high feed costs and great challenges for aquaculture. Although plant proteins have wide source and low prices, the existence of anti-nutrient factors greatly limits their large-scale application in aquafeed [1]. Among plant proteins, soybean meal is the most widely used plant protein source [2]. Previous studies have reported that the anti-nutrient factors in soybean meal, such as protease inhibitors, soybean agglutinin, and oligosaccharides, negatively affected the growth [3], intestinal health [4] and immunity [5] of aquatic animals. Therefore, it is necessary to develop new methods to degrade the anti-nutrient factors of soybean meal.

Fermentation can degrade the anti-nutrient factors and macromolecular nutrients in soybean meal, and it can increase bioactive components such as peptides and soybean isoflavones [6]. Yeast culture is also a yeast-fermented product, which is composed of yeast cells, metabolites and fermented medium formed by yeast through multi-stage fermentation [7]. Yeast culture contains plenty of β-glucan and mannan oligosaccharide, which can promote the growth, intestinal health and immunity of aquatic animals [8]. It has been reported that yeast culture successfully substituted for 40 g/kg fishmeal without a significant impact on the growth and immunity of Pacific white shrimp (*Litopenaeus vannamei*) fed a diet with 200 g/kg fishmeal inclusion [7]. The replacement of 20 g/kg fishmeal with *Saccharomyces cerevisiae* culture effectively improved the growth performance and feed utilization of channel catfish (*Ictalurus punctatus*) [9]. Furthermore, partially replacing fishmeal with yeast culture has been observed in largemouth bass (*Micropterus salmoides*) [10], gibel carp (*Carassius auratus gibelio*) [11], and freshwater prawn (*Macrobrachium rosenbergii*) [12].

Yellow catfish (*Pelteobagrus fulvidraco*) is a freshwater fish widely distributed in China and other Asian countries. This fish has been extensively cultured in China for its substantial economic and nutritional value with good taste. Yellow catfish is a carnivorous fish, thus the consumption of fishmeal and animal proteins in commercial feeds is relatively high. Exploring low fishmeal diets has far-reaching significance for the healthy development of yellow catfish aquaculture. Chen et al. [13] reported that the substitution of fishmeal with yeast culture improved the growth and immunity of yellow catfish. However, in that study, the yeast culture inclusion was only 20 g/kg; obviously, such a inclusion level is very low for a protein ingredient. Therefore, yeast culture was used as an alternative protein source to reduce graded levels of fishmeal to evaluate the effects on the growth performance, nutrient utilization, serum biochemical indices, and intestinal and hepatopancreas histology of yellow catfish. The findings will explore the potential of yeast culture as an alternative protein source in aquatic feeds.

## 2. Materials and Methods

### 2.1. Ethics Statement

All the procedures for handling the animals involved in this experiment are in accordance with the regulations of the Experimental Animal Ethics Committee and the Institutional Animal Care Committee of Shanghai Ocean University (Approval code: SFI 2020-23 of 20 May 2020).

### 2.2. Experimental Design

First, a basal diet was designed as the control with the inclusion of 160 g/kg fishmeal (Con). Then, yeast culture was used to reduce the fishmeal level to 120, 80, 40, 0 g/kg (YC-12, YC-8, YC-4, and YC-0). After crushing, sieving (80-mesh) and mixing, the ingredients in the mixture were extruded to form sinking pellets with a 2 mm diameter by a single-screw extruder (LX-75 Extruder, Longxiang Food Machinery Factory, Baoding, China). Then, the diets were air-dried and stored at 4 °C. The ingredients and proximate composition of the experimental diets are shown in Table 1.

The yeast culture used in this study was provided by Shanghai Yuanyao Agricultural Co., Ltd. (Shanghai, China), and it is a yeast-fermented product with enzymatic hydrolyzed soybean meal as the culture substrate. After the fermentation, the yeast cells were inactivated by increasing the temperature. The mannan and nucleotide contents of the yeast culture were 22.0 g/kg and 2.01 g/kg, respectively. The proximate composition and amino acid profiles of the yeast culture and fishmeal are shown in Table 2.

### 2.3. Experimental Plan

Yellow catfish were purchased from a local farm in Huzhou (Zhejiang, China). After two weeks of stocking, a total of 600 yellow catfish (3.00 ± 0.10 g) were randomly divided into 15 polyvinyl tanks (diameter 1.0 m, height 0.8 m, water volume 650 L), with three replicates per treatment and 40 fish per replicate (tank). All the tanks shared the same circulating and filtering system, with a flowing speed of 10 L/min for each tank. Yellow catfish were hand-fed at 9:00 and 16:00, and the daily feeding amount was about 3–5% of the body weight, which was appropriately adjusted to ensure no feed residue. The feces was removed by siphoning every day. During the feeding period, the dissolved oxygen, ammonia content, nitrite content, and water temperature were 6.5 ± 0.5 mg/L, 0.13 ± 0.06 mg/L, 0.07 ± 0.03 mg/L, and 25 ± 2 °C, respectively. The breeding process was carried out at Binhai Aquaculture Station (Shanghai Ocean University, China) for 56 days.

### 2.4. Sample Collection

Before the feeding trial, 20 fish were selected from the initial population to measure the whole-body composition. After 56 days of feeding, the number and weight of all the fish in each tank were measured to calculate the weight gain (WG), feed conversion ratio (FCR), specific growth rate (SGR), survival rate (SR) and feed intake (FI). After the anaesthetization, three fish were randomly selected from each tank to measure the whole-body composition, while another three fish per tank were used to measure the body length and body weight to calculate the condition factor (K). Subsequently, the blood was drawn from the cordial vein, and the supernatant was sampled after centrifugation (3500 r/min, 10 min, 4 °C) to freeze at −80 °C for the measurement of the serum biochemical indicators. Then, the fish were dissected and the corresponding tissues were sampled to determine the hepatosomatic index (HSI) and viscerosomatic index (VSI). Hepatopancreas (0.25 cm^2^ × 1 cm) and intestine (about 1/3 of foregut) samples were cut off and stored in Bouin’s solution for tissue sections. The remaining foregut was used to measure the enzyme activity (amylase, trypsin, lipase, and DAO) of the intestinal tissue.

### 2.5. Measurement and Methods

#### 2.5.1. Growth Performance and Physical Indices

The below Formula are the calculations for the SR, WG, FCR, SGR, K, VSI, HSI and FI:

SR (%) = 100 × (the final fish number/the initial fish number)

WG (%) = 100 × [final weight (g) − initial weight (g)]/initial weight (g)

FCR = feed consumption (g)/weight gain (g)

SGR (%BW/day) = 100 × [ln final weight (g) − ln initial weight (g)]/days

K (g/cm^3^) = 100 × [final body weight (g)/final body length^3^ (cm)^3^]

VSI (%) = 100 × [visceral weight (g)/final body weight (g)]

HSI (%) = 100 × [hepatopancreas weight (g)/final body weight (g)]

FI (g/fish/d) = feed intake (g)/[(final fish number + initial fish number)/2]/days

#### 2.5.2. Composition of the Whole-Body and Diet 

According to the approach of the AOAC [14], the moisture content was measured by drying the sample at 105 °C in an oven. The levels of crude ash, crude protein and crude lipid were determined using the burning method (550 °C), Kjeldahl method (2300 Auto analyzer, Foss, Copenhagen, Denmark) and chloroform–methanol extraction method, respectively. The amino acid composition in the diet was pretreated according to the method described by Huang et al. [1], and then the samples were detected and analyzed by automatic amino acid analyzer (S-433D, Sykam, Munich, Germany).

#### 2.5.3. Serum Biochemical Indices

The serum indices were measured by commercial kits (Nanjing Jiancheng Biotechnology Co., Ltd., Nanjing, China), including aspartate aminotransferase (AST), alanine aminotransferase (ALT), triglyceride (TG), glucose (GLU), total cholesterol (TCHO), total protein (TP), malondialdehyde (MDA), catalase (CAT), superoxide dismutase (SOD), and alkaline phosphatase (AKP). D-lactic acid (D-LA), immunoglobulin M (IgM) and complement 3 (C3) were measured by ELISA kits supplied by the same producer.

#### 2.5.4. Nutrient Retention

The nutrient utilization indices are the calculations for the PER, PR and LR:

PER (protein efficiency ratio, %) = 100 × [final body weight (g) − initial body weight (g)]/protein intake (g)

PR (protein retention, %) = 100 × [final body weight (g) × crude protein content of the final fish − initial body weight (g) × crude protein content of the initial fish]/protein intake (g)

LR (lipid retention, %) = 100 × [final body weight (g) × crude lipid content of the final fish − initial body weight (g) × crude lipid content of the initial fish]/lipid intake (g)

#### 2.5.5. Intestinal Digestive Enzyme and Intracellular Enzyme Activity

The thawed intestinal tissue (0.1 g) was homogenized with normal saline (1:9 *w*/*v*) in an ice bath and then centrifuged at 2500 r/min for 10 min (4 °C) to determine the intestinal digestive enzymes activity with the supernatant [15].

According to the description included in the kit, trypsin can catalyze the hydrolysis of the ester chain of the substrate arginine ethyl ester at pH 8.0 and 37 °C, and the activity of the enzyme can be determined according to the change in its absorbance at 253 nm, and trypsin in one milligram of protein changing the absorbance by 0.003 per minute was defined as one unit (U/mg prot). The blue complex is formed by the combination of iodine solution and starch, and amylase can hydrolyze starch, which is used to calculate the activity of amylase by comparing the change in the absorbance [16], and 10 mg starch hydrolyzed by one milligram of tissue protein at 37 °C for 30 min was defined as one unit (U/mg prot). The activities of diamine oxidase (DAO) and lipase were measured by kits (Nanjing Jiancheng Biotechnology Co., Ltd., Nanjing, China).

#### 2.5.6. Intestinal and Hepatopancreas Histology

The foregut and hepatopancreas tissues were dehydrated in a series of alcohol, equilibrated, embedded, sliced (RM2235, Leica, Nussloch, Germany) and stained. Photographs were taken of the sections with a microscope (YS100, Nikon, New York, NY, USA), and software (Image J14.0) was used to measure the villus heights, villus width and muscle thickness. All the methods referred to Zhao et al. [17].

### 2.6. Statistical Analysis

All the data were expressed as the mean ± standard deviation (mean ± SD), and then a one-way ANOVA was conducted (SPSS 26.0 software). The statistical differences between the groups were determined by Duncan’s multiple test (*p* < 0.05).

## 3. Results

### 3.1. Growth Performance and Physical Indices

In Table 3, the SR of the yellow catfish was 100% during the feeding period. There was no significant difference in the WG, FCR, and SGR between the YC-12 group and the CON group (*p* > 0.05). Compared with the CON group, the WG in the YC-8, YC-4 and YC-0 groups was decreased (−16.6%, −21.2%, and −26.1%), and the FCR in the YC-4 and YC-0 groups was increased (+0.30 and +0.50) (*p* < 0.05). There was no significant difference in the K, VSI, and HSI among the five groups (*p* > 0.05).

### 3.2. Whole-Body Composition and Nutrient Utilization

In Table 4, only the YC-0 group showed a significantly lower crude lipid content than the CON group (*p* < 0.05). There were no significant differences in the contents of moisture, crude ash, and crude protein among the groups (*p* > 0.05). Compared with the CON group, the PER and PR in the YC-8, YC-4 and YC-0 groups, as well as the LR in the YC-4 and YC-0 groups, were significantly decreased (*p* < 0.05). 

### 3.3. Serum Biochemical Indices

In Table 5, the YC-0 group showed significantly higher AST and ALT activity, and significantly lower TCHO content, than the CON group (*p* < 0.05). Compared with the CON group, the CAT and SOD activities in the YC-12 and YC-8 groups were significantly increased, and the MDA content was decreased significantly (*p* < 0.05). No significant difference was detected in the serum contents of TG, GLU and TP among all the groups (*p* > 0.05).

In terms of the serum immune indices, the contents of C3 and IgM in the YC-12 and YC-8 groups, as well as the AKP activity in the YC-12, YC-8, and YC-4 groups, were significantly higher than those in the CON group (*p* < 0.05). The D-lactic acid content presented a significantly lower value in the YC-12 group and a significantly higher value in the YC-0 group than the CON group (*p* < 0.05).

### 3.4. Intestinal Digestive Enzyme and Intracellular Enzyme Activity

Compared with the CON group, the trypsin activity in the YC-12 and YC-8 groups, as well as the DAO activity in the YC-4 and YC-0 groups, were significantly increased (*p* < 0.05), while there was no significant difference in the amylase and lipase activities among all the groups (*p* > 0.05) (Table 6). 

### 3.5. Intestinal Morphology

**Figure 1:**
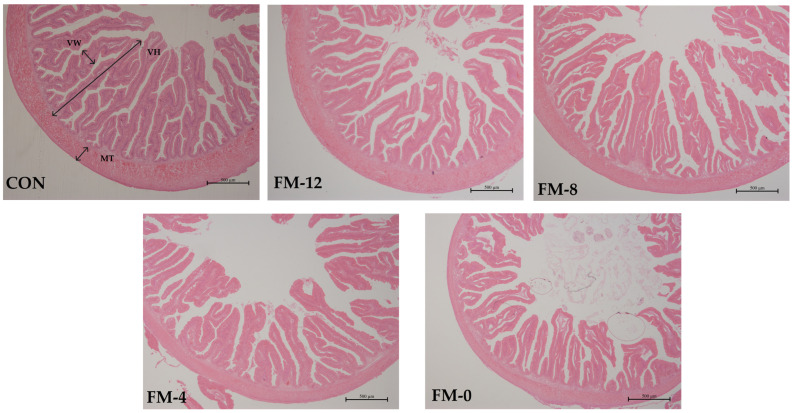
Effects of replacing fishmeal with yeast culture on the intestinal morphology of yellow catfish (4×). VH: villus height; VW: villus width; MT: muscular thickness. Scale bar = 500 μm.

The intestinal morphology is shown in Figure 1 and Table 7. The villus height of the YC-4 and YC-0 groups and the villus width of the YC-0 group were significantly lower than those of the CON group (*p* < 0.05). There was no significant difference in muscular thickness among all the groups (*p* > 0.05).

### 3.6. Hepatopancreas Morphology

**Figure 2:**
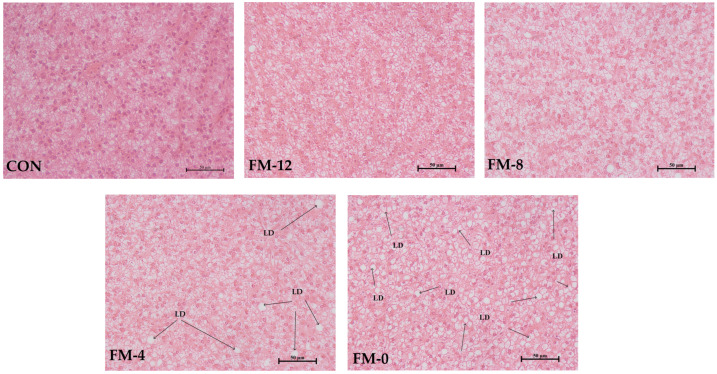
Effects of replacing fishmeal with yeast culture on the hepatopancreas morphology of yellow catfish (40×). LD: lipid droplet. Scale bar = 50 μm.

As shown in Figure 2, lipid droplets appeared in the YC-4 and YC-0 groups, and severe cell vacuolation was observed in the YC-0 group.

## 4. Discussion

### 4.1. Growth Indices

Previous studies have verified the beneficial effects of dietary yeast culture on the growth and immunity of aquatic animals. Ayiku et al. [18] once used yeast culture (1% or 2%) to iso-nitrogenously reduce the dietary fishmeal inclusion, and they found that the growth performance, immunity, antioxidant and intestinal health of Pacific white shrimp were enhanced by the substitution. Yeast culture has been also reported to effectively reduce the fishmeal inclusion from 350, 250, 200 g/kg to 250, 130, and 160 g/kg in the diets of largemouth bass [19], pacu (*Piaractus mesopotamicus*) [20] and Pacific white shrimp [7]. The present study demonstrated that the substitution of 40 g/kg fishmeal with yeast culture did not negatively affect the growth of yellow catfish.

However, when the substitution of fishmeal with yeast culture was ≥80 g/kg, negative impacts were observed on the growth and feed efficiency of yellow catfish (Table 3). The WG and PER were significantly reduced when the fishmeal replaced by yeast culture reached 120 g/kg (the basal diet contained 200 g/kg fishmeal) in *Litopenaeus vannamei* [7]. Similarly, when 150 g/kg fishmeal was replaced by yeast culture (350 g/kg of fishmeal in the diet), the WG and nutrient utilization of largemouth bass were also significantly decreased [19]. The reduced growth performance and nutrient utilization by the high substitution of fishmeal with yeast culture could be elucidated as follows. (1) Lack of some essential amino acids such as methionine (Table 2). The yeast culture used in this study is a fermented product with enzymatic soybean meal, thus the yeast culture presents similar amino acids profiles to soybean, with relatively lower methionine content. Jiang et al. [21] supplemented lysine and methionine to a high soybean meal diet and found that the growth performance of yellow catfish was improved and the intestinal damage was alleviated. In addition, methionine is involved in the synthesis of taurine in vivo, which can promote the growth and development of fish [22]. (2) Yeast culture is a plant-origin product and lacks some bioactive compounds rich in fishmeal, including taurine, hydroxyproline, cholesterol and some unidentified growth factors. Insufficient taurine intake would result in a decline in both growth performance and immunity. The supplementation of taurine in all-plant protein diets significantly improved the growth performance and resistance against ammonia nitrogen stress of yellow catfish [23]. (3) Yeast culture contains plenty of mannose and β-glucan (important components of the yeast cell wall), thus the increase in mannose and β-glucan may aggravate the expression of immune mechanism, resulting in insufficient energy and nutrition for growth [24].

The present investigation revealed that the substitution of fishmeal with yeast culture did not adversely affect the content of moisture, crude protein, and crude ash of yellow catfish, which was consistent with previous studies conducted in Pacific white shrimp [25] and largemouth bass [19]. However, the crude lipid content in the YC-0 group was significantly decreased, which may be related to the decrease in TCHO in the serum. Yeast culture has also been reported to enhance the population of lactobacilli and bifidobacterial in the intestine, which have the ability to assimilate cholesterol and regulate cholesterol synthesis through the utilization of fermentation products [24].

### 4.2. Serum Biochemical and Immune Indices

AST and ALT are two transaminases that reflect the health of the liver. The increasing serum activities of AST and ALT usually mean the damage of liver function [26]. In this study, the serum AST and ALT activities increased in correlation with the increase in yeast culture and the decrease in fishmeal inclusion. Notably, the AST and ALT activities in the YC-0 group were significantly increased compared to those in the CON group, and the vacuolization in the liver cells was significantly increased, which indicated that the liver function of the YC-0 group was adversely impacted. In gibel carp [11], when the proportion of yeast culture replacing fishmeal was more than 60%, the activities of AST and ALT were also increased significantly. In general, the production and elimination of reactive oxygen species in the body are in a dynamic equilibrium. Once the balance is destroyed, fish will suffer from oxidative stress and even disease [27]. Generally, CAT and SOD serve as the primary defense mechanisms against oxidative stress in fish, facilitating the elimination of peroxides. The assessment of the MDA level is commonly employed to evaluate the degree of peroxidation in the body. MDA exhibits potent toxicity to impair cellular structure and function [28]. In this study, the SOD and CAT activities in the YC-12 and YC-8 groups were significantly higher, and the MDA content was significantly lower, than in the CON group, indicating that the replacement of 40 and 80 g/kg fishmeal by yeast culture improved the antioxidant capacity of yellow catfish. Yeast culture contains plenty of β-glucan and mannan oligosaccharides originating from the yeast cell wall, which have been verified in terms of the immunity-promoting effects in many studies. Bai et al. [29] reported that the isomeric hydrogen in β-glucan can scavenge free radicals. Dietary supplementation of β-glucan (4–8 g/kg) improved the activity of antioxidant enzymes and reduced the content of MDA in the intestine and liver of tilapia (*Oreochromis niloticus*) [30]. Lu et al. [31] found that adding 1.5 g/kg β-glucan increased the CAT activity of Nile tilapia, which was consistent with the results in *Macrobrachium rosenbergii* [32]. The supplementation of mannan oligosaccharides (0.2–0.6 g/kg) to feed also significantly improved the intestinal antioxidant capacity and intestinal health of grass carp (*Ctenopharyngodon idella*) [33]. Furthermore, Gu et al. [34] confirmed that β-glucan and mannan oligosaccharides had a synergistic effect, and the combined supplementation resulted in the highest SOD activity for sea cucumber (*Apostichopus japonicus*).

In this study, the contents of the complementary C3, IgM and AKP activity in the YC-12 and YC-8 groups were significantly higher than in the CON group, indicating that dietary yeast culture improved the non-specific immune function of yellow catfish. Such promoting effects have also been reported in Pacific white shrimp [8] and channel catfish [9]. β-glucan can bind to specific receptors on the surface of phagocytes, then improve the phagocytic activity and antibacterial ability of phagocytes, and promote cell proliferation and stimulate the production of new phagocytes [35]. Mannan oligosaccharides can occupy the receptor of pathogens in the intestine and reduce the adhesion of pathogens [36]. It has been reported that dietary supplementation of β-glucan-rich chrysophyte (*Poterioochromonas malhamensis*) (20–40 g/kg) [37] or β-glucan and mannan oligosaccharides mixture (20–30 g/kg) [38] improved intestinal health, immunity and disease resistance of rainbow trout and common carp (*Cyprinus carpio*). However, the excessive β-glucan content led to immune fatigue for white shrimp [39], which may be the reason for the decrease in immunity in the YC-0 group.

### 4.3. Intestinal Digestive Enzyme and Intestinal Morphology

Intestinal digestive enzyme activity and morphology are important reference indexes reflecting intestinal health. The metabolites produced by yeast culture during fermentation, including peptides, umami nucleotides and oligosaccharides, can promote the reproduction and vitality of beneficial microflora and improve the intestinal digestion and absorption capacity [40]. In this study, the trypsin activity in the YC-12 and YC-8 groups was significantly increased by the inclusion of yeast culture in the diets. Wang et al. [41] once reported that the supplementation of yeast culture (40 g/kg) to the diet significantly increased the trypsin activity of hybrid grouper. A similar result was also found in sea bass (*Dicentrarchus labrax*) [42].

The villus height and width determine the contact area of nutrient digestion and absorption. The muscular thickness promotes intestinal peristalsis and digestion [18]. The increase in the diamine oxidase activity and D-lactic acid content is the result of an intestinal permeability change, which is usually used as an indicator for assessing intestinal damage [43,44]. In this study, the villus height in the YC-4 and YC-0 groups and the villus width in the YC-0 group were significantly reduced, and the diamine oxidase activity and D-lactic acid content in the YC-4 and YC-0 groups were significantly increased, indicating that intestinal structure was damaged and intestinal inflammation was induced when a high level of fish meal was substituted by yeast culture. The occurrence of intestinal inflammation may be related to the decrease in the antioxidant capacity. Chen et al. [45] found that metabolites produced by oxidative stress destroyed intestinal structural barriers, increased intestinal permeability and led to enteritis. In addition, the decrease in the intestinal villus height and width may be related to the decrease in bioactive substances such as taurine due to the low inclusion of fishmeal. The supplementation of taurine (8 g/kg) to the low fishmeal diet significantly increased the villus height and intestinal muscle layer thickness of *Trachinotus ovatus* [46].

## 5. Conclusions

In a practical diet containing 160 g/kg fishmeal, yeast culture can effectively replace 40 g/kg fishmeal without negatively affecting the growth, nutrient utilization, serum immune and antioxidant, intestinal and hepatopancreas histology of yellow catfish.

## Figures and Tables

**Table 1:** Ingredients and proximate composition of the experimental diets (air-dried basis, g/kg).

Ingredients ^1^	CON	YC-12	YC-8	YC-4	YC-0
Fishmeal	160	120	80	40	0
Yeast culture	0	53.5	107	160.5	214
Soybean meal	230	230	230	230	230
Soybean protein concentrate	60	60	60	60	60
Wheat flour	286	268.8	251.7	234.5	217.3
Corn gluten meal	50	50	50	50	50
Chicken meal	100	100	100	100	100
Meat meal	50	50	50	50	50
Fish oil	0	3.7	7.3	11	14.7
Soybean oil	24	24	24	24	24
Soybean lecithin	10	10	10	10	10
Ca(H_2_PO_4_)_2_	20	20	20	20	20
Vitamin and mineral premix ^2,3^	10	10	10	10	10
Total	1000	1000	1000	1000	1000
Proximate composition (g/kg)					
Moisture	95.3	80.3	76.6	73.2	88.1
Crude protein	384.9	388.5	387.0	392.8	387.7
Crude lipid	74.8	73.1	68.1	67.9	69.0
Crude ash	75.1	74.3	72.0	66.8	65.5

^1^ Among the fishmeal, soy bean meal, wheat flour, chicken meal, and meat meal, the protein content was 672.2, 442.0, 144.1, 654.2, and 740.0 g/kg, respectively. ^2^ Vitamin (mg or IU/kg diet): VA, 6000 IU; VD_3_, 2000 IU; VE, 50 mg; VK_3_, 6 mg; VB_1_, 10 mg; VB_2_, 10 mg; VB_6_, 8 mg; VB_12_, 0.02 mg; VC, 150 mg; biotin, 0.2 mg; inositol, 100 mg; and folic acid, 2 mg; D-calcium pantothenate, 30 mg. ^3^ Mineral (mg/kg diet): I, 1.0 mg; Mn, 10 mg; Co, 1 mg; Cu, 5 mg; Zn, 60 mg; Se, 0.35 mg; and Fe, 80 mg.

**Table 2:** The proximate composition and amino acid profiles of the yeast culture and fishmeal (air-dried basis, g/kg).

Items	Yeast Culture	Fishmeal
Proximate composition (air-dried basis)		
Crude protein	550.0	672.2
Crude lipid	16.4	104.0
Crude ash	65.3	149.5
Phosphorus	7.4	28.5
Essential amino acids (dry-matter basis)		
Lysine	33.7	50.6
Methionine	7.4	19.4
Arginine	40.2	38.9
Histidine	16.3	16.0
Isoleucine	24.5	30.8
Leucine	44.1	50.0
Phenylalanine	29.6	27.5
Threonine	24.8	28.1
Tryptophan	6.7	7.6
Valine	25.8	34.8
Non-essential amino acids (dry-matter basis)		
Aspartic acid	55.3	62
Serine	25.9	29.2
Glutamic acid	96.2	89.5
Glycine	23.1	37.4
Alanine	25.8	44.2
Cysteine	3.5	6.1
Proline	37.5	28.4
Tyrosine	20.2	23.8
Total amino acids	540.6	624.3

**Table 3:** Effects of substituting fishmeal with yeast culture on the growth of yellow catfish.

Items	CON	YC-12	YC-8	YC-4	YC-0
IBW (g)	3.08 ± 0.04	3.05 ± 0.05	3.08 ± 0.08	3.08 ± 0.09	3.03 ± 0.07
FBW (g)	19.13 ± 0.35 ^a^	18.46 ± 0.59 ^ab^	16.57 ± 1.18 ^bc^	15.75 ± 1.00 ^c^	14.91 ± 0.69 ^c^
WG (%)	524.45 ± 8.88 ^a^	505.17 ± 9.34 ^a^	437.25 ± 34.98 ^b^	412.54 ± 36.49 ^b^	386.86 ± 20.46 ^b^
FCR	1.37 ± 0.04 ^c^	1.43 ± 0.07 ^bc^	1.59 ± 0.14 ^bc^	1.67 ± 0.06 ^ab^	1.87 ± 0.15 ^a^
FI (g/fish)	22.08	22.08	22.08	22.08	22.08
SGR (%/d)	3.27 ± 0.03 ^a^	3.21 ± 0.03 ^ab^	3.00 ± 0.11 ^bc^	2.92 ± 0.13 ^c^	2.82 ± 0.08 ^c^
SR (%)	100	100	100	100	100
K (g/cm^3^)	1.59 ± 0.06	1.61 ± 0.11	1.68 ± 0.05	1.66 ± 0.04	1.68 ± 0.05
VSI (%)	7.32 ± 0.78	7.49 ± 0.60	8.12 ± 0.80	8.09 ± 0.29	7.88 ± 0.34
HSI (%)	1.31 ± 0.20	1.30 ± 0.11	1.31 ± 0.07	1.37 ± 0.28	1.12 ± 0.02

In the same row, values with different small letter superscripts mean significant differences (*p* < 0.05). IBW: initial body weight; FBW: final body weight; WG: weight gain; FCR: feed conversion ratio; FI: feed intake; SGR: specific growth rate; SR: survival rate; K: condition factor; VSI: viscerosomatic index; HSI: hepatosomatic index.

**Table 4:** Effects of substituting fishmeal with yeast culture on the whole-body composition and nutrient utilization of yellow catfish.

Items	CON	YC-12	YC-8	YC-4	YC-0
Moisture	732.4 ± 4.7	730.4 ± 7.2	739.7 ± 11.4	743.1 ± 11.1	747.9 ± 7.8
Crude ash	35.1 ± 1.6	36.8 ± 4.3	34.2 ± 1.8	35.4 ± 3.7	35.8 ± 2.9
Crude lipid	56.1 ± 5.9 ^a^	53.6 ± 5.3 ^ab^	59.1 ± 3.7 ^a^	49.8 ± 3.8 ^ab^	44.2 ± 1.3 ^b^
Crude protein	142.3 ± 2.3	142.8 ± 4.4	137.7 ± 3.6	141.5 ± 1.1	138.1 ± 2.8
PER	2.00 ± 0.06 ^a^	1.85 ± 0.09 ^a^	1.50 ± 0.03 ^b^	1.53 ± 0.05 ^b^	1.39 ± 0.11 ^b^
PR (%)	29.38 ± 0.84 ^a^	27.31 ± 1.32 ^a^	22.52 ± 1.87 ^b^	21.59 ± 1.64 ^b^	21.05 ± 2.25 ^b^
LR (%)	60.69 ± 1.65 ^a^	56.95 ± 2.65 ^a^	57.78 ± 1.37 ^a^	49.36 ± 1.72 ^b^	42.47 ± 4.18 ^c^

In the same row, values with different small letter superscripts mean significant differences (*p* < 0.05). PER: protein efficiency ratio; PR: protein retention; LR: lipid retention.

**Table 5:** Effects of substituting fishmeal with yeast culture on the serum immune and antioxidant of yellow catfish.

Items	CON	YC-12	YC-8	YC-4	YC-0
AST (U/mL)	2.25 ± 0.22 ^b^	2.29 ± 0.33 ^b^	2.52 ± 0.01 ^ab^	2.60 ± 0.22 ^ab^	3.11 ± 0.63 ^a^
ALT (U/mL)	1.58 ± 0.18 ^b^	1.71 ± 0.39 ^ab^	1.82 ± 0.09 ^ab^	1.98 ± 0.12 ^ab^	2.10 ± 0.09 ^a^
TG (mmol/L)	1.63 ± 0.04	1.61 ± 0.07	1.45 ± 0.14	1.42 ± 0.09	1.58 ± 0.08
GLU (g/L)	1.56 ± 0.11	1.51 ± 0.09	1.48 ± 0.12	1.45 ± 0.06	1.44 ± 0.02
TCHO (mmol/L)	3.21 ± 0.44 ^a^	3.11 ± 0.14 ^a^	2.74 ± 0.22 ^ab^	2.35 ± 0.67 ^ab^	1.77 ± 0.48 ^b^
TP (g/L)	35.73 ± 0.48	36.03 ± 3.80	35.73 ± 5.20	34.16 ± 2.11	34.10 ± 3.60
MDA (nmol/mL)	4.04 ± 0.13 ^a^	2.33 ± 0.79 ^b^	2.70 ± 0.31 ^b^	3.14 ± 0.81 ^ab^	3.89 ± 0.23 ^a^
CAT (U/mL)	8.06 ± 0.22 ^b^	9.92 ± 0.61 ^a^	9.67 ± 1.28 ^a^	8.91 ± 0.63 ^ab^	8.63 ± 0.28 ^ab^
SOD (U/mL)	101.14 ± 5.61 ^b^	117.37 ± 3.15 ^a^	122.52 ± 6.96 ^a^	115.88 ± 5.64 ^a^	113.34 ± 10.36 ^ab^
C3 (μg/mL)	60.03 ± 13.45 ^b^	78.17 ± 1.76 ^a^	82.33 ± 7.68 ^a^	70.68 ± 6.11 ^ab^	61.36 ± 2.89 ^ab^
IgM (μg/mL)	19.58 ± 3.57 ^c^	24.77 ± 0.61 ^ab^	28.26 ± 1.01 ^a^	22.05 ± 3.40 ^bc^	21.00 ± 1.41 ^bc^
AKP (U/L)	40.32 ± 1.22 ^a^	50.67 ± 3.64 ^b^	53.26 ± 0.61 ^b^	49.91 ± 6.17 ^b^	46.70 ± 1.98 ^ab^
D-LA (μmol/L)	6.42 ± 0.08 ^b^	3.93 ± 0.40 ^c^	6.63 ± 0.42 ^b^	7.68 ± 0.20 ^b^	13.59 ± 1.58 ^a^

In the same row, values with different small letter superscripts mean significant differences (*p* < 0.05).

**Table 6:** Effects of substituting fishmeal with yeast culture on the intestinal digestive enzyme and intracellular enzyme of yellow catfish.

Items	CON	YC-12	YC-8	YC-4	YC-0
Amylase (U/mg prot)	0.25 ± 0.02	0.25 ± 0.03	0.28 ± 0.04	0.30 ± 0.04	0.28 ± 0.01
Trypsin (U/mg prot)	3542.81 ± 50.97 ^b^	4382.74 ± 350.65 ^a^	4677.12 ± 303.35 ^a^	4223.82 ± 46.24 ^ab^	3975.84 ± 129.93 ^ab^
Lipase (U/mg prot)	142.33 ± 13.35	142.94 ± 21.33	163.03 ± 14.00	147.95 ± 11.76	158.02 ± 1.82
DAO (U/mg prot)	11.11 ± 1.63 ^b^	11.64 ± 2.18 ^ab^	13.65 ± 1.21 ^ab^	14.21 ± 0.69 ^a^	14.28 ± 1.52 ^a^

In the same row, values with different small letter superscripts mean significant differences (*p* < 0.05).

**Table 7:** Effects of substituting fishmeal with yeast culture on the intestinal morphology of yellow catfish.

Items (μm)	CON	YC-12	YC-8	YC-4	YC-0
Villus height	1766.1 ± 139.5 ^a^	1759.8 ± 119.2 ^a^	1703.4 ± 250.1 ^a^	1477.7 ± 196.3 ^b^	1183.3 ± 138.8 ^c^
Villus width	306.0 ± 24.7 ^a^	306.3 ± 31.7 ^a^	307.1 ± 49.1 ^a^	272.8 ± 43.3 ^ab^	252.0 ± 25.4 ^b^
Muscular thickness	307.7 ± 43.7	293.1 ± 29.3	258.7 ± 60.5	259.2 ± 28.3	264.1 ± 22.5

In the same row, values with different small letter superscripts mean significant differences (*p* < 0.05).

## Data Availability

The data involved in this paper can be found in the article.
